# Structural, Physicochemical, and Functional Properties of Wheat Bran Insoluble Dietary Fiber Modified With Probiotic Fermentation

**DOI:** 10.3389/fnut.2022.803440

**Published:** 2022-05-04

**Authors:** Ai-Mei Liao, Jie Zhang, Zhen-Lin Yang, Ji-Hong Huang, Long Pan, Yin-Chen Hou, Xiao-Xiao Li, Peng-Hui Zhao, Yu-Qi Dong, Zhe-Yuan Hu, Ming Hui

**Affiliations:** ^1^School of Biological Engineering, Henan University of Technology, Zhengzhou, China; ^2^School of Food and Pharmacy, Xuchang University, Xuchang, China; ^3^Henan Provincial Key Laboratory of Biological Processing and Nutritional Function of Wheat, Henan University of Technology, Zhengzhou, China; ^4^College of Food and Biological Engineering, Henan University of Animal Husbandry and Economy, Zhengzhou, China; ^5^Henan Cooperativity Medical Science and Technology Research Institute Co., Ltd, Luoyang, China; ^6^Henan Provincial Engineering Laboratory of Preservation and Breeding of Industrial Microbial Strains, Henan University of Technology, Zhengzhou, China

**Keywords:** insoluble dietary fiber, biological modification, physicochemical property, functional properties, antioxidant activity

## Abstract

Insoluble dietary fiber (IDF) were isolated from wheat bran (WB) after microbial fermentation with single or mixed strain [*Lactobacillus plantarum, Lactobacillus acidophilus, Bacillus subtilis* or mixed lactic acid bacteria (*L. plantarum* and *L. acidophilus* with ration of 1:1)]. Structure, physicochemical, functional properties, and antioxidant activity of the wheat bran insoluble dietary fiber (W-IDF) modified by fermentation were studied. Fourier transformed infrared spectroscopy (FT-IR), and scanning electron microscopy (SEM) analysis suggested the successful modification of W-IDF. After fermentation with *L. plantarum* and mixed lactic acid bacteria, the water retention capacity (WRC), oil retention capacity (ORC), and water swelling capacity (WSC) of W-IDF were improved. The sodium cholate adsorption capacity (SCAC), and cation exchange capacity (CEC) of W-IDF modified with *L. acidophilus* fermentation were significantly increased. Although the cholesterol adsorption capacity (CAC) of W-IDF decreased after modification with probiotic fermentation, nitrite ion adsorption capacity (NIAC), and total phenolic content (TPC) were enhanced. Additionally, W-IDF modified by fermentation with *B. subtilis* or mixed lactic acid bacteria exhibited superior antioxidant capacity verified by DPPH, ABTS and total reducing power assays. Results manifested that microbial fermentation is a promising methods to modify the W-IDF to provide high-quality functional IDF for food processing and human health management.

## Introduction

In modern diet, daily intake of dietary fiber (DF) is inadequate for most people, due to the development of refined foods and excessive energy intake. Correspondingly, the incidence of sub-health and modern civilization diseases has been increased. Reasonable intake of DF is critical for health since it has been recognized as a beneficial nutrient to improve digestive tract health, lower blood lipids, regulate blood glucose, and control body weight. DF is generally divided into soluble dietary fiber (SDF) and insoluble dietary fiber (IDF). IDF displayed crucial role on human health via promoting intestinal peristalsis, improving bowel movements, protecting digestive system, reducing energy density, adsorbing and discharging grease and toxic substances such as heavy metals (1, 2). Additionally, supplement of IDF demonstrated a significant impact on the processing characteristics, sensory quality, and stability of various food products (3). Consequently, beneficial effects of IDF on management of human health and food industry has become more attractive since the average intake of total DF and IDF of adult residents in China is deficient.

It was indicated that the various functional effects performed by IDF closely do with their different structures and components. Various methods have been applied to modify the structure or properties of IDF, such as physical, chemical, enzymatic or combined methods, and microbial fermentation. After modification, properties of IDF including hydration, adsorption, ion exchange activities were accordingly improved, which made IDF have a stronger ability to suppress the activity of enzymes including lipase and digestive enzymes (4–6). Therefore, the expansion of high-quality IDF supplement channels or the favorable effects of IDF on human health and food characteristics during processing have attracted more and more attention in related research (7).

Wheat bran (WB), a kind of main by-products of flour processing, consists of abundant IDF, yet its utilization efficiency is low because of the poor palatability and difficulty in digestion of wheat bran IDF (W-IDF) (8). Modification of WB has been carried out to change its technological properties, improve its healthy benefits, or facilitate the preparation processes of foods such as bread (9). Fermentation has been regarded as a potential method of producing high-quality DF, playing an important role in improving its physicochemical properties or functional activities, such as WRC, WSC, ORC, and anti-diabetic effect (10, 11). It was suggested that modification of millet bran DF through fermentation with *Bacillus natto* not only enhanced the proportion of SDF, but also increased the adsorption capacity of IDF for cholesterol, bile salts, nitrite, as well as glucose. The content of total phenol and DPPH free radical scavenging ability were also visibly improved (12). Fermentation with yeast in combination with enzyme settlement of WB in whole-wheat bread obviously enhanced the bioavailability of phenolic acids, contributing to its anti-inflammatory influence *ex vivo* (13, 14). However, there are few reports on the effect of biological modification on the physicochemical, and functional properties of W-IDF.

In present study, W-IDF was biologically modified by fermentation with single or mixed strain (*L. plantarum, L. acidophilus, B. subtilis*, and mixed *L. plantarum, L. acidophilus* with ration of 1:1). Effects of biological modification on the structural, physicochemical, functional properties, and antioxidant activity of W-IDF were also evaluated, which will provide a new effective method to obtain functional W-IDF to extend its potential application in fields of food processing, and development of functional food.

## Materials and Methods

### Materials

WB was purchased from Henan Hebi Feitian Agricultural Development Co., Ltd, α- Amylase (≥3,700 U/g) was purchased from Aoboxing Biotechnology Co., Ltd (Beijing, China). Papain (600,000 U/g) and ABTS (≥98% ) were purchased from Solarbio Co., Ltd (Beijing, China). DPPH (96%) was purchased from Macklin Co., Ltd (Shanghai, China). Strains: *L. acidophilus, L. plantarum*, and *B. subtilis* were all preserved in the laboratory. The chemicals and reagents used are of analytical grade.

### Modification of Wheat Bran Dietary Fiber

WB was crushed after impurity removal treatment, autoclaved at 115°C for 30 min. After cooling to room temperature, activated microbial strains (*B. subtilis, L. acidophilus, L. Plantarum*, or mixed lactic acid bacteria that was *L. Acidophilus : L. Plantarum* = 1:1) were inoculated at a feed liquid ratio of 1:1 with amount of 5% (~10^8^ CFU/ml). They were stirred then followed by fermentation at 37°C for 48 h.

After fermentation, the untreated and microbial-fermented WB were dried at 60°C to constant weight, sieved through 60 mesh sieve after superfine crushing, then degreased with petroleum ether at the material liquid ratio of 1:20 (g/ml), shaking for 4 h at 40°C. After filtration, the precipitate was washed. After drying, the obtained WB was kept at −20°C for further analysis.

### Extraction of Insoluble Dietary Fiber

The defatted WB was hydrolyzed with α-amylase (0.02 g/g) at 55°C for 2 h with the condition as following: pH 6.5, material-to-liquid ratio of 1:20 (g/ml). After the hydrolysis, the precipitate was washed with deionized water (DW) for three times, then dried at 70°C to a constant weight.

The dried sample without starch was hydrolyzed with papain (0.002 g/g) at 40°C for 1.5 h with pH of 6, material-to-liquid ratio of 1:5 (g/ml). After the enzymolysis, the fermentation substances were heated in a water bath at 100°C for 5 min to inactivate, then the precipitate was washed with DW for three times and dried at 70°C to a constant weight. Afterwards, decoloration was carried out with 8% H_2_O_2_ at 70°C for 4 h, followed by washing the products with DW until neutral and dried at 70°C to obtain different kinds of W-IDF modified with various microbial fermentation which were a blank control group IDF (C-IDF), *L. plantarum* IDF (P-IDF), *L. acidophilus* IDF (A-IDF), *B. subtilis IDF* (B-IDF), mixed lactic acid bacteria IDF (M-IDF), respectively.

### Structural Characterization

#### Fourier-Transformed Infrared Spectroscopy

Fourier-Transformed Infrared Spectroscopy (FT-IR) analysis was carried out to measure the structure of the W-DIF with Use Nicolet iS20 (Thermo Scientific). The infrared spectrum was recorded in the range of 650–4,000 cm^−1^, 32 scans, and the resolution was 0.25 cm^−1^.

#### Scanning Electron Microscopy

The IDF were examined using scanning electron microscopy (SEM) (FEI Quanta 250, US) at 3 KV. Installed the powder sample on a metal platform with conductive tape and sputter-coated with gold. Images of each sample were taken at 2,000 × magnification.

### Physicochemical and Functional Properties

#### Loose Density

1.0 g of IDF (m) was added into a 25 ml graduated cylinder at a free falling speed through a funnel with a diameter of 6 cm, measuring its volume as V_1_. The loose density (LD) was measured as the following formula calculation.


$$\text{LD}\;(\text{g}/\text{ml})\;=\;\frac{\text{m}}{{\text{V}}_{1}}$$

#### Tapped Bulk Density

1.0 g of IDF (m) was poured into a 25 ml graduated cylinder at a free-falling speed through a funnel with a diameter of 6 cm. A wooden board was fixed on the shaker so that the funnel could hit the board, and then the shaker was reciprocated at 120 r/min for 2 h. The resulting volume was the bulk volume V_2_, which was then calculated according to the following formula.


$$\text{TBD}\;(\text{g}/\text{ml})\;=\;\frac{\text{m}}{{\text{V}}_{2}}$$

#### Water Retention Capacity

Refer to the method of Esposito et al. (15), 2.5 g of IDF (m_1_) was put into a 50 ml centrifuge tube (m_0_). It was kept at room temperature for 30 min after adding 30 ml of DW. The supernatant was discarded after centrifugation at 2,500 r/min for 10 min, recording the total mass of the centrifuge tube and insoluble dietary fiber as m_2._ The water retention capacity (WRC) was calculated according to the following formula.


$$\text{WRC}\;(\text{g}/\text{g})\;=\;\frac{{\text{m}}_{2}\;-\;{\text{m}}_{0}\;-\;{\text{m}}_{1}}{{\text{m}}_{1}}$$

#### Oil Retention Capacity

According to the method described by Liu et al. (16) with modification. 1.0 g IDF (m_1_) was kept in a 50 ml centrifuge tube (m_0_). After adding 20 g soybean oil, the centrifuge tube was placed at 37°C for 1 h, followed by centrifuging at 3,000 r/min for 20 min. After removing the upper oil, the total mass of the centrifuge tube and IDF was recorded as m_2_, and then the oil retention capacity (ORC) was calculated by the following formula.


$$\text{ORC}\;(\text{g}/\text{g})\;=\;\frac{{\text{m}}_{2}\;-\;{\text{m}}_{0}\;-\;{\text{m}}_{1}}{{\text{m}}_{1}}$$

#### Water Swelling Capacity

Based on the method of Ma and Mu (17), 0.5 g of IDF (m) was weighted and placed into a 10 ml graduated cylinder. The volume of unhydrated IDF in the graduated cylinder was recorded as V_1_. 5.0 ml of DW was accurately measured and added. The IDF was suspended in the added DW for 24 h at room temperature. The volume of hydrated IDF was marked as V_2_. Then the water swelling capacity (WSC) was calculated according to the following formula.


$$\text{WSC}\;(\text{ml}/\text{g})\;=\;\frac{{\text{V}}_{2}\;-\;{\text{V}}_{1}}{\text{m}}$$

#### Cation Exchange Capacity

The method provided by Carvalho et al. (18) with slight modification was applied to analyze cation exchange capacity (CEC). 15 ml of 0.1 mol/L HCl solution was added to 0.50 g of WDF. The solution was stirred well and kept at room temperature for 24 h. After filtration, the sample was rinsed until the filtrate didn't contain Cl^−^. The filtrate was checked with a 10% AgNO_3_ solution, and titrated until there was no white precipitate, which proved that the chloride ions have been removed. The filter residue was collected and placed in a drying oven at 60°C with blowing air for drying. 0.30 g of the dried sample was dispersed in 100 ml 15% NaCl solution, and stirred magnetically for 5 min. After adding phenolphthalein (2 g/L) for color development, the solution with was titrated with 0.1 mol/L sodium hydroxide to a light red color, recording the consumption of sodium hydroxide as V_1_ (ml), hydroxide as V_2_ (ml), then the consumption volume of sodium hydroxide as V_1_-V_2_.


$$\text{CEC}\;(\text{m mol}/\text{g})\;=\;\frac{0.1\times ({\text{V}}_{1}\;-\;{\text{V}}_{2})}{\text{m}}$$

Where V_1_, V_2_ and m was represented as the titration volume of the sample (ml), the titration volume of the blank (ml) and the dietary fiber quality (g), respectively.

#### Sodium Cholate Adsorption Capacity

As per the previous method Kahlon and Chow (19), 25 ml of 2 mg/ml sodium cholate was added to a 150 ml Erlenmeyer flask containing 0.5 g IDF. The pH of the solution was adjusted to 6.0 with a citric acid-sodium citrate buffer solution, followed by magnetically stirring until the dispersion was uniform. Then the solution was kept at 37°C with medium speed of shaking for 2 h. 1.0 ml of the supernatant was drew out and determined the concentration of sodium cholate for calculating the sodium cholate adsorption capacity (SCAC) of IDF.


$$\text{SCAC}\;(\text{mg}/\text{g})\;=\;\frac{{\text{m}}_{1}\;-\;{\text{m}}_{2}}{\text{m}}$$

Where m_1_, m_2_ and m represented the sodium cholate content before adsorption (mg), the sodium cholate content after adsorption (mg) and the dietary fiber quality (g), respectively.

#### Cholesterol Adsorption Capacity

On the basis of the method employed by Zhang et al. (20), yolk of a fresh egg was taken and stirred into an emulsion with 9 times DW. Each 50 g of diluted egg yolk was transferred to 200 ml Erlenmeyer flasks with 1.0 g of IDF, followed by adjusting the pH value of the system to 2.0 and 7.0, respectively, as imitative gastroenteric environments. Solutions were shaked at 37°C for 2 h before centrifuging at 4,000 r/min for 20 min. 1 ml of the supernatant was diluted five times with 90% glacial acetic acid. After adequately mixturing, 0.1 ml of the diluted solution was measured the cholesterol content of the supernatant at 550 nm with o-phthalaldehyde as the developer.


$$\text{CAC}\;(\text{mg}/\text{g})\;=\;\frac{{\text{m}}_{1}\;-\;{\text{m}}_{2}}{\text{m}}$$

Where m_1_, m_2_, and m represent the cholesterol content before adsorption (mg), the cholesterol content after adsorption (mg), and the dietary fiber quality (g), respectively.

#### Nitrite Ion Adsorption Capacity

Following the method described by Zhu et al. (21), each 1.0 g IDF in the 250 ml Erlenmeyer flasks was added 100 ml of NaNO_2_ solution with a concentration of 1 mmol/L. pH of the systems were adjusted to 2.0 and 7.0, respectively, and kept at 37°C for 2 h with magnetic stirring 0.1 ml of sample solution was applied to determine the ${\text{NO}}_{2}^{-}$ content according to the naphthalene ethylenediamine hydrochloride method, which determined the amount of nitrite adsorbed by the difference between the amount of nitrite before and after the reaction.


$$\text{NIAC}\;(\mu \text{mol}/\text{g})\;=\;\frac{{\text{n}}_{1}\;-\;{\text{n}}_{2}}{\text{m}}$$

Where n_1_, n_2_, and m represent the nitrite content before adsorption (μmol), the nitrite content after adsorption (μmol) and the dietary fiber quality (g), respectively.

### Antioxidant Properties

#### Extraction of Antioxidant Substance

Refer to the method reported by Zhang et al. (22) with slight modification, 1 g of dried IDF was placed in the 15 ml centrifuge tube. After adding 10 ml of 80% ethanol solution, the solution was transferred to a 70°C water bath for 2 h, then the solution was performed ultrasonic-assisted extraction at 70°C for 10 min before cooling to room temperature. After centrifugation at 5,000 r/min for 10 min, the supernatant was collected, wraped in tin foil, and stored at 4°C for further analysis.

#### Determination of Total Phenolic Content

Based on the method introduced by Dong et al. (23), 5 ml of 10% Folin was added to 1 ml of gallic acid working solution of different concentrations (0, 12.5, 25, 50, 100, 200, 400 μg/ml) with shaking for 5 min to mixture the solution completely. Once 4.0 ml 7.5% Na_2_CO_3_ solution was added, the solution was placed in the dark at room temperature for 60 min. The absorbance at 765 nm was measured, then a standard curve was set up. Measure the absorbance of the sample solution in the same way and calculate the total phenolic content (mg/g).


$$\text{TPC}\;(\text{mg}/\text{g})\;=\;\frac{\text{C}\;\times \;\text{V}\;\times \;\text{D}}{\text{m}}$$

Where C, V, D, and m represent the total phenol concentration (mg/ml), the extract volume (ml), the dilution times, and the dietary fiber quality (g), respectively.

#### DPPH Radical Scavenging Capacity

According to the method described by Xu and Chang (24), the obtained antioxidant substance was diluted five times with DW. 100 μl of the diluted solution was placed in a 96-well plate, then 100 μl of DPPH-absolute ethanol solution (100 μmol/L) was added and gently mixed. The 96-well plate was kept in the dark at room temperature for 30 min. The absorbance was measure at 517 nm and marked as A_1_, Replacing the sample solution with absolute ethanol to determine the absorbance as A_0_. Substituting the DPPH-ethanol solution with absolute ethanol to measure the absorbance value (A_2_).


$$\text{DPPH}\;(\text{I}\text{\%})\;=\;\frac{{\text{A}}_{0}\;-\;{\text{A}}_{1}\;+\;{\text{A}}_{2}}{{\text{A}}_{0}}\times 100\text{\%}$$

#### ABTS Radical Scavenging Capacity

As per Re et al. (25) with slight modification, equal volume of 7.4 mmol/L ABTS and 2.6 mmol/L potassium persulfate solution were mixed and reacted in the dark for 12–16 h, The solution was diluted with DW to make the absorbance of the solution 0.70 ± 0.02 at 734 nm to prepare a stock solution of ABTS free radicals.

The prepared antioxidant substance mentioned above was diluted five times with DW. 0.9 ml of ABTS free radical stock solution was added to 100 μl of the diluted solution, and mixed well, followed by keeping in the dark at room temperature for 5 min. Its OD value was determined at 734 nm (A_1_). The sample solution was replaced with absolute ethanol to determine the OD value (A_0_), while replacing the ABTS free radical stock solution with DW to determine the OD value (A_2_).


$$\text{ABTS}\;(\text{I}\text{\%})\;=\;\frac{{\text{A}}_{0}\;-\;{\text{A}}_{1}\;+\;{\text{A}}_{2}}{{\text{A}}_{0}}\times 100\text{\%}$$

#### Total Reducing Power

Refer to the previous study Aryal et al. (26), the antioxidant substance mentioned above was diluted five times with DW. 2 ml of the sample solution was added with 2 ml 0.2 mol/L phosphate buffer (pH = 6.6), 2 ml (1%, w/v) potassium ferricyanide and mixed the solution. The system was reacted in a water bath at 50°C for 20 min, then immediately cooled to room temperature. 2 ml of trichloroacetic acid solution (10%, w/v) was employed to stop the reaction. After centrifugation at 3,500 rpm for 10 min, 2 ml of the supernatant was mixed with 2 ml of DW and 0.4 ml of FeCl_3_ solution, and kept in the dark at room temperature for 10 min. The OD value at 700 nm was measured. The reducing power was measured by the absorbance value of the sample solution at 700 nm.

### Statistical Analysis

Three parallel experiments were performed for each group of experiments, and the average value and standard deviation were calculated. The experimental data were expressed as “mean ± standard deviation” (Mean ± SD). Origin 2021, SAS 9.2 were used for drawing and ANOVA difference analysis.

## Results

### Structural Characterization

#### FT-IR

**Figure 1:**
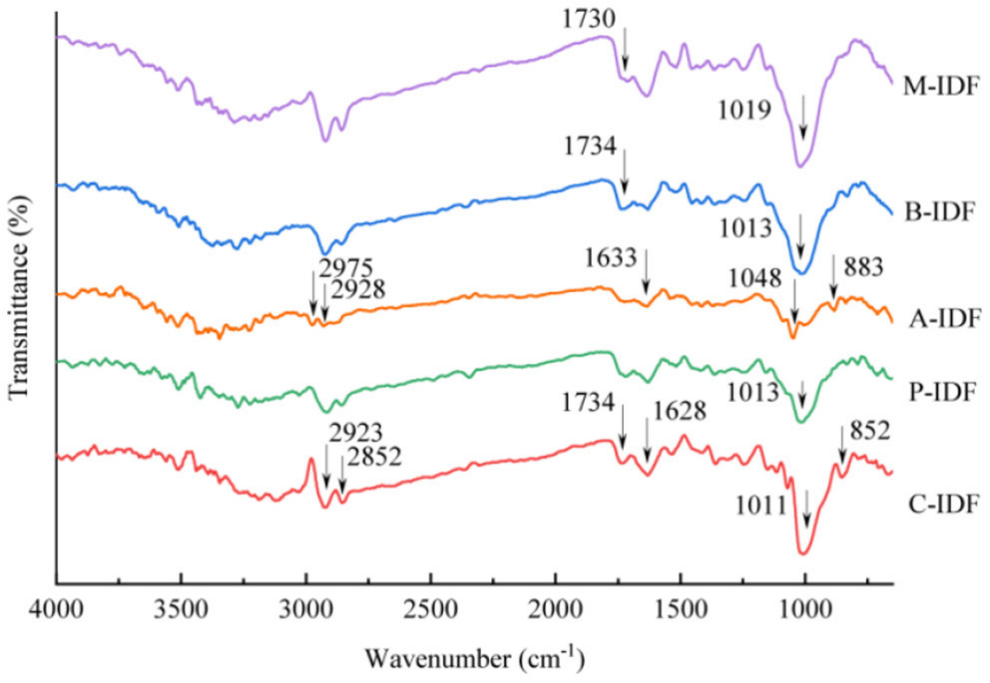
FT-IR spectra of wheat bran insoluble dietary fiber (W-IDF) modified with different microbial. C-IDF, W-IDF without microbial modification; P-IDF, W-IDF modified by *L. plantarum*; A-IDF, W-IDF modified by *L. acidophilus*; B-IDF, W-IDF modified by *B. subtilis*. M-IDF, W-IDF modified by mixed lactic acid bacteria (*L. acidophilus: L. plantarum* = 1:1).

FT-IR analysis was applied to characterize five kinds of modified W-IDF as shown in Figure 1. The peak intensity and width of A-IDF at 2,920 cm^−1^ was significantly reduced compared with C-IDF. Honcu et al. (27) found that the band around this was related to the C-H vibration of methyl and methylene groups. The destruction of the methyl and methylene components of the hydrogen bond can reduce the peak intensity and width. The bands at 2,858.38 and 1,743.56 cm^−1^ represent the C-H vibrations of some methylene groups in polysaccharides and carbonyl groups, respectively (28). The peaks at 1,634.91 and 1,024.15 cm^−1^ were corresponded to characteristic bending or stretching of carboxyl groups, which are interconnected with cellulose chains by forming intermolecular hydrogen bonds (17).

These peaks all appeared in the spectrum, but their intensities in A-IDF were all lower than C-IDF, indicating that some of the hydrogen bonds between the cellulose chains were broken. In addition, Zhao et al. (28) found that the peak at 810.61 cm^−1^ indicated the stretching vibration of the β-glycosidic bond in polysaccharides. In P-IDF and M-IDF, it was almost undetectable, indicating that the fermentation with *L. plantarum* and mixed lactic acid bacteria can destroy the hydrogen bonds of intermolecular cellulose and hemicellulose and β-glycosides of pectin.

#### SEM

**Figure 2:**
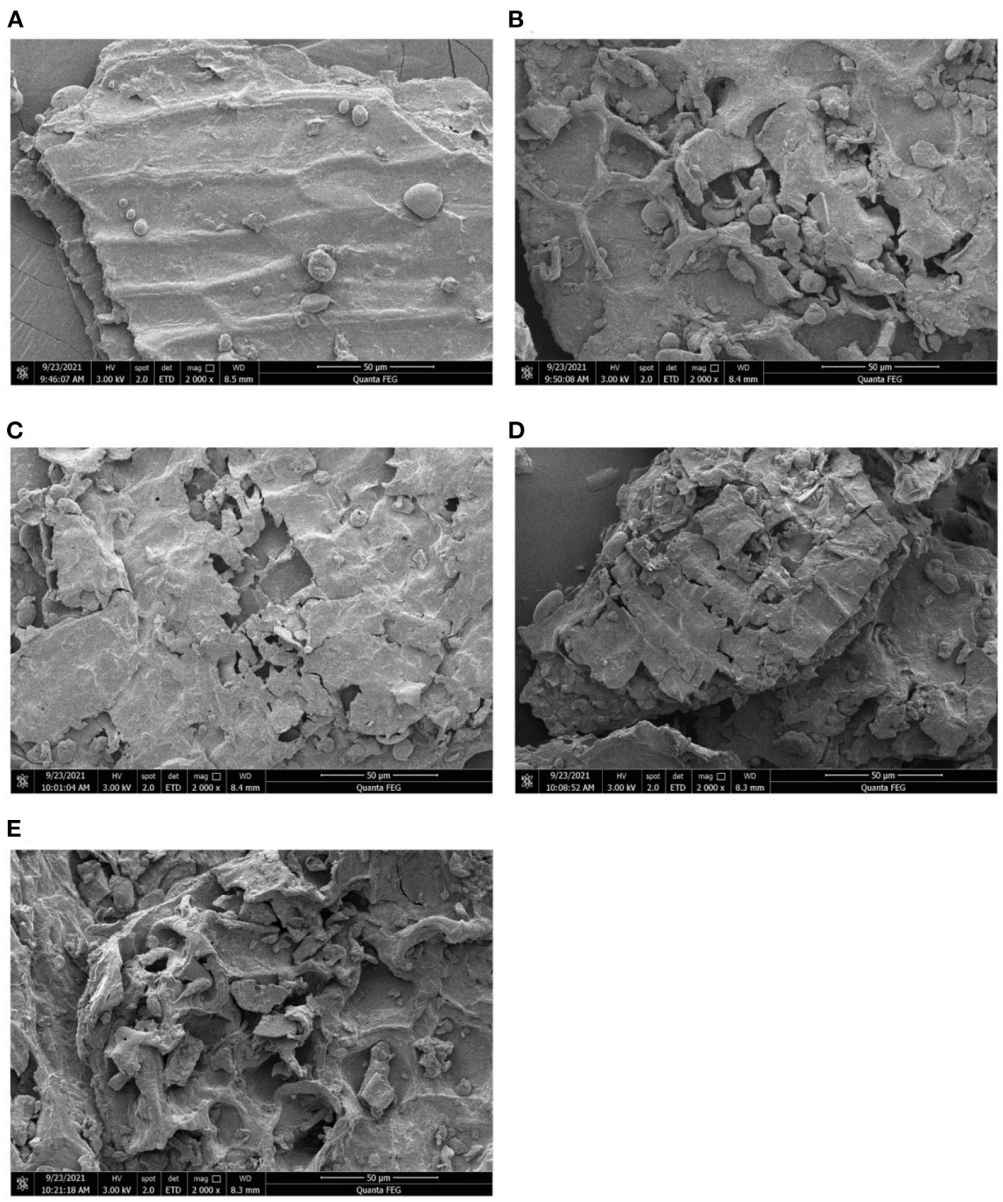
Scanning electron microscopy (SEM) image of W-IDF. **(A)** C-IDF, **(B)** P-IDF, **(C)** A-IDF, **(D)** B-IDF, **(E)** M-IDF.

The scanning electron microscope (SEM) images of the modified and unmodified W-IDF were shown in Figure 2. C-IDF (Figure 2A) had a smooth surface and a relatively complete wall structure. After microbial fermentation and modification, W-IDF showed great differences. Among them, the surfaces of A-IDF (Figure 2C) and B-IDF (Figure 2D) were loose, with holes and cracks, which may due to the degradation of wall polysaccharides and the destroyed wall structure (22). On the other hand, P-IDF (Figure 2B) and M-IDF (Figure 2E) indicated more pores and cracks in the honeycomb structure, and the structural integrity was damaged.

### Physicochemical Properties

**Figure 3:**
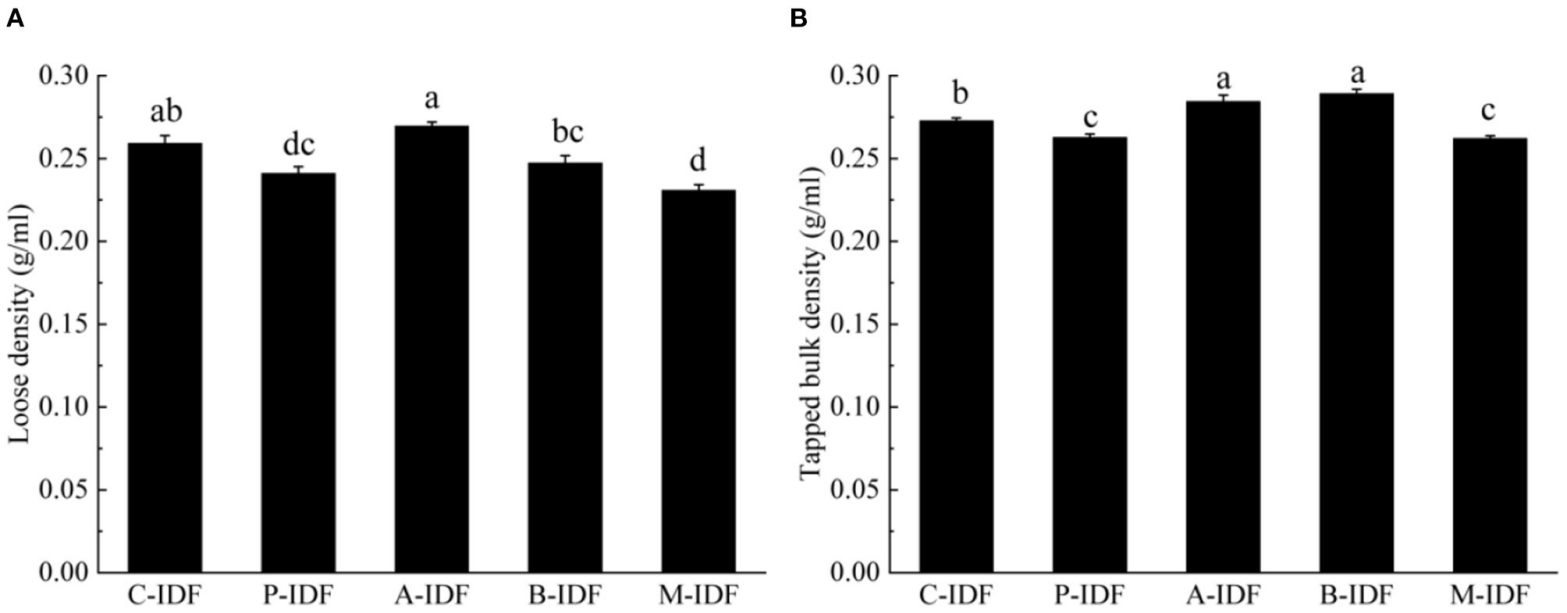
Density of W-IDF. **(A)** Loose density (LD), **(B)** tapped bulk density (TBD). Data were expressed as means ± SD with different letters representing significant difference (*p* < 0.05).

**Figure 4:**
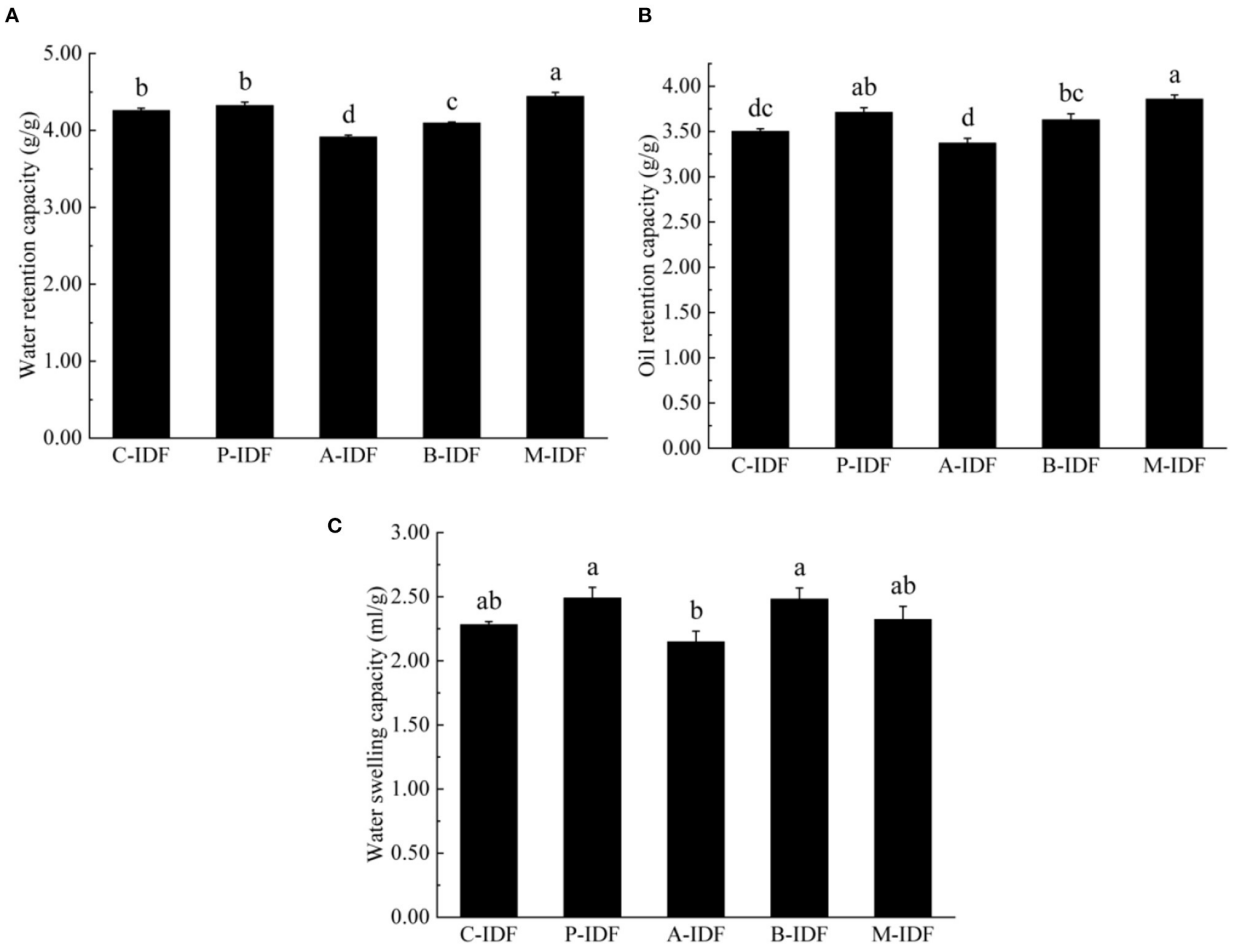
Physicochemical properties of W-IDF. **(A)** Water retention capacity (WRC), **(B)** oil retention capacity (ORC), **(C)** water swelling capacity (WSC). Data were expressed as means ± SD with different letters representing significant difference (*p* < 0.05).

After modification with microbial fermentation, the altered physical property of W-IDF were demonstrated in Figures 3, 4. As shown in Figure 3, the density of M-IDF (0.231 g/ml) and P-IDF (0.241 g/ml) was lower than that of C-IDF (0.259 g/ml) (*p* < 0.05). As shown in Figure 4A, the WRC of M-IDF (4.44 g/g) and P-IDF (4.33 g/g) were higher than C-IDF (3.92 g/g), and M-IDF was significantly higher than that of the blank group (*p* < 0.05). The ORC of M-IDF (3.86 g/g) and P-IDF (3.71 g/g) were also significantly higher compared to the one of C-IDF (3.50 g/g) (*p* < 0.05) (Figure 4B). As shown in Figure 4C, the WSC of P-IDF and M-IDF was improved.

### Adsorption Functional Properties

**Figure 5:**
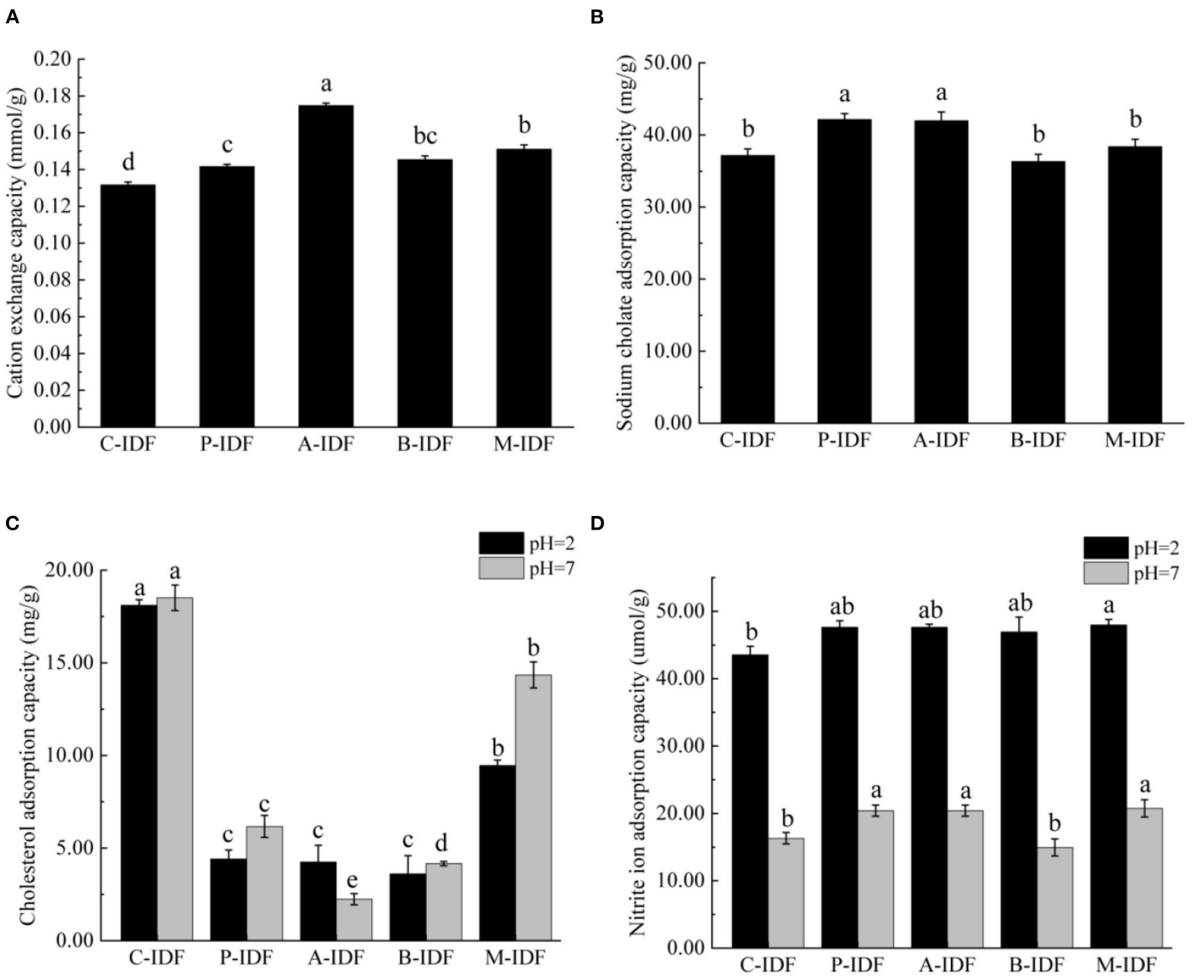
Functional adsorption capacity of W-IDF. **(A)** Sodium cholate adsorption capacity (SCAC), **(B)** Cholesterol adsorption capacity (CAC), **(C)** Cation exchange capacity (CEC), **(D)** Nitrite ion adsorption capacity (NIAC). Data were expressed as means ± SD with different letters representing significant difference (*p* < 0.05).

Compared with C-IDF (0.132 mmol/g), the cation exchange capacity (CEC) of fermentation modified group was significantly improved (*p* < 0.05) (Figure 5A). Among them, A-IDF (0.175 mmol/g) displayed the most significant increase, which was 1.33 times that of C-IDF. This may be due to the strong acid-producing ability of *L. acidophilus*, which has a greater impact on the titration of the adsorbed cholesterol solution.

The adsorption capacity of IDF for sodium cholate (SCAC) was shown in Figure 5B. SCAC of P-IDF (42.17 mg/g), and A-IDF (41.97 mg/g) was significantly enhanced in comparison to the one of C-IDF (37.18 mg/g) (*p* < 0.05). The modified P-IDF and A-IDF's ability to bind bile acid was improved, which not only greatly reduced the raw materials for synthesizing cholesterol, but also directly reduces the cholesterol content in the body.

Cholesterol adsorption of capacity (CAC) of W-IDF at different pH was summarized in Figure 5C. Under pH 2 (simulating the environment of human gastric juice) C-IDF (18.51 mg/g) and pH 7 (simulating the environment of human small intestine) C-IDF (18.11 mg/g) revealed a significantly higher CAC than other groups (*p* < 0.05). Except for A-IDF, CAC at pH 7 was greater than the one at pH 2, indicating that the acidity and alkalinity of the system also had a greater impact on the ability of DF to adsorb cholesterol.

As shown in Figure 5D, the pH of the reaction system performed a very significant impact on the ability of W-IDF to adsorb nitrite ion (${\text{NO}}_{2}^{-}$) (NIAC) at pH 7 (simulating the environment of the human small intestine). The NIAC of P-IDF (20.40 μmol/g), A-IDF (20.40 μmol/g), and M-IDF (20.74 μmol/g) was higher than that of C -IDF (37.18 μmol/g) (*p* < 0.05). For all W-IDF, NIAC at pH 2 (simulating human gastric juice environment) was significantly stronger than that under pH 7 environment.

### Antioxidant Properties

**Figure 6:**
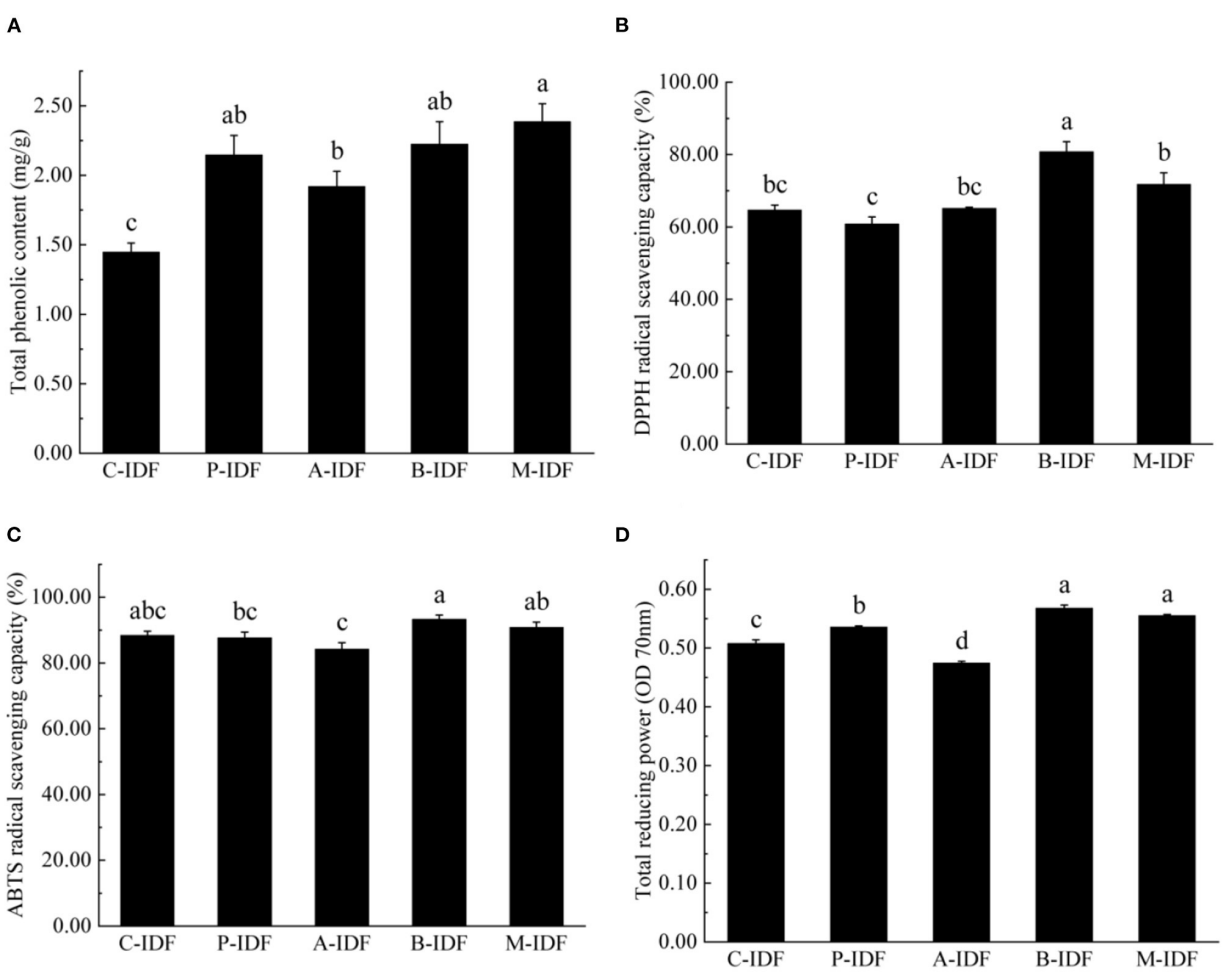
Antioxidant activity of W-IDF. **(A)** Total phenolic content (TPC), **(B)** DPPH radical scavenging capacity, **(C)** ABTS radical scavenging capacity, **(D)** total reducing power. Data were expressed as means ± SD with different letters representing significant difference (*p* < 0.05).

The determination results of total phenolic content (TPC) for different IDF were summarized in Figure 6A. Compared with C-IDF (1.45 mg/g), each group after microbial fermentation indicated a significant increase of TPC (*p* < 0.05). Among them, M-IDF (2.39 mg/g) revealed the largest TPC, which was 1.65 times that of C-IDF.

As shown in Figure 6B, the DPPH radical scavenging rate of B-IDF (80.79%), M-IDF (71.77%), and A-IDF (65.16%) were all greater than that of C-IDF (64.70%), and B-IDF. Compared with DPPH, the free radical scavenging rate of C-IDF was significantly increased (*p* < 0.05).

As shown in Figure 6C, there was no significant difference between IDF and C-IDF (88.42%) after fermentation modification. The ABTS free radical scavenging rate of B-IDF was the highest at 93.3%, which was 15.5% higher than that of DPPH free radical scavenging power (80.79%), exhibiting superior ABTS free radical scavenging ability. As shown in Figure 6D, the reducing power of B-IDF (0.57), M-IDF (0.56), and P-IDF (0.54) were significantly higher than C-IDF (0.47) (*p* < 0.05).

## Discussion

W-IDF was biologically altered by fermentation with single or multi-strains. Fermentation effects on the structure, physicochemical nature, and functional activities, and antioxidant abilities of W-IDF were also investigated in present research. The modified IDF and C-IDF were basically similar in spectra except for differences in absorbance and/or wavelength in some characteristic bands. Results of FT-IR characterization demonstrated that biological modification with *L. acidophilus* was stronger than the one with other three. This may result from its strong acid production ability, which exerts a great impact on the arrangement of chemical bonds of W-IDF. After fermentation modification, structural changes possibly arised from the enzymatic hydrolysis that removed the protein or starch around the IDF. Meanwhile, the acid production of lactic acid bacteria caused degradation to cellulose and hemicellulose (29). This might mount the specific surface area of IDF, thereby conducing to improve its adsorption and binding capacity (30, 31).

After fermentation with bacilli and mixed lactic acid bacteria, WRC and ORC of IDF were significantly increased. Damage of cellulose and hemicellulose mentioned above lead to enhanced SDF content, as a result, the WRC was arised. SDF content was suggested to be positively related to WRC in a previous study (30). Increase of ORC may because of surface features, hydrophobic characteristics of the IDF particle. IDF with higher WSC added in food can enhance the satiety of consumers, reduce the intake of other foods, and help prevent obesity. The improvement of WRC and ORC is conducive to better functional properties of DF in human body, promotes the peristalsis of the small intestine, and contributes to the development of food with lower glycemic index in the later period. Functional groups in side chains of IDF such as carboxyl, hydroxyl, and amino groups may exist a function, which is similar to a weakly acidic cation exchange resin, reversibly exchange with organic cations, and change the instantaneous concentration of ions (32). Chau and Huang (33) found that CEC of IDF mainly dependents on carboxyl, and hydroxyphenolic groups which were speculated to be affected by microbial fermentation modification to modulate CEC of IDF.

Generally, SCAC and CAC are considered to conduct the ability of DF to lower the level of blood pressure and blood lipid and the potential application of DF to develop functional foods preventing again cardiovascular health. After modification the SCAC of IDF became greater. Additionally, the modified IDF displayed higher SCAC and CAC at pH 7 than those at pH 2. Consistent observation was suggested by previous research for cocoa shells DF (34). The ability to reduce the nitrite ion was possibly related to the phenolic acids in DF and the pH environment. Møller et al. (35) believed that the active ingredient of W-DF to remove ${\text{NO}}_{2}^{-}$ is phenol. In acidic condition, ferulic acid (accounting for 75–90% of the total phenolic content) is the only phenol that can effectively react with ${\text{NO}}_{2}^{-}$. Also, Lignin (IDF) is a phenolic polymer that can react with nitrite under acidic condition, usually at pH ≤ 2 and high temperature (70°C). When the pH increases, the carboxyl group in compounds (uronic acid, ferulic acid, etc.) dissociate, which increases the negative charge density on the surface of DF, thus repelling ${\text{NO}}_{2}^{-}$ and releasing it (36).

Antioxidant properties of biological modified W-IDF was investigated *via* TPC, DPPH, and ABTS assay. Antioxidant ability is passably concerned with the structure and composition of IDF especially phenolic acid. WB mainly consists of two types of phenolic acid, cinnamic acid, benzoic acid, and p-hydroxybenzoic acid, which can be divided into alulic acid, ferulic acid, cafieic acid, vanillin, p-hydroxybenzoic acid, eugenic acid, and so on (22). Among them, ferulic acid, ferulic acid, vanillic acid, and p-hydroxybenzoic acid are major extractable phenolic acids (37). TPC of the modified IDF was increased probably due to the release of phenolic acids in the bran during fermentation. DPPH radical scavenging ability was demonstrated to be positively correlated with total antioxidant capacity. The scavenging ability of IDF on DPPH free radicals after fermentation can indicate the strength of its antioxidant capacity (23). Previous investigations have suggested that antioxidant capacity of WB is closely connected with the increase of ferulic acid-based polyphenols released by fermentation (38). ABTS free radical is a positively charged free radical. It can obtain an electron from the antioxidant molecule to form a stable neutral molecule. Therefore, elimination of ABTS free radical mainly involves the electron transfer mechanism. The level of reducing power indicated the electronic donating ability of the antioxidant substance, which is a significant indicator for the antioxidant capacity of sample. The stronger the reducing power, the stronger the antioxidant ability of the sample (23). Savolainen et al. (39) revealed that fermentation can release the bound phenols in WB, thereby causing changes in antioxidant activity. Antioxidant activities of IDF modified by biological fermentation in present study was increased due to the enhancement of TPC resulted from the release of bound ferulic acid. Antioxidant activities of the IDF can increase cellular defense and contribute to prevent cellular components against oxidative damage.

## Conclusion

In this study, fermentation with a variety of probiotics was employed to modify wheat bran dietary fiber (W-DIF), which improved the physical, chemical, and functional properties of IDF. After fermentation by *L. plantarum* and mixed lactic acid bacteria, WRC, ORC, and WSC of W-IDF were improved. After fermentation by *L. acidophilus*, the SCAC, and CEC of W-IDF were significantly increased. NIAC, and TPC of W-IDF were heightened after fermentation and modification by probiotics, though the CAC of IDF was reduced. Meanwhile, B-IDF and M-IDF have revealed superior antioxidant capacity. Microbial fermentation is a promising approach to vary the structure, physicochemical, functional properties, and antioxidant activity of W-IDF. W-DIF fermented and modified by mixed lactic acid bacteria is a higher-quality nutrient with extensive potential application in field of food processing and development of functional food to satisfy the public demand for healthy food.

## Data Availability Statement

The original contributions presented in the study are included in the article/supplementary material, further inquiries can be directed to the corresponding author/s.

## Author Contributions

A-ML: formal analysis, investigation, data curation, writing-original draft, and funding acquisition. JZ: investigation, data curation, and writing-original draft. Z-LY, X-XL, P-HZ, Y-QD, and Z-YH: investigation and data curation. LP: software and formal analysis. Y-CH: data curation and writing-review. MH: writing-review and editing. J-HH: visualization, project administration, writing-review and editing, funding acquisition, and supervision. All authors contributed to the article and approved the submitted version.

## Conflict of Interest

Y-CH was employed by Henan Cooperativity Medical Science and Technology Research Institute Co., Ltd. The remaining authors declare that the research was conducted in the absence of any commercial or financial relationships that could be construed as a potential conflict of interest.

## Publisher's Note

All claims expressed in this article are solely those of the authors and do not necessarily represent those of their affiliated organizations, or those of the publisher, the editors and the reviewers. Any product that may be evaluated in this article, or claim that may be made by its manufacturer, is not guaranteed or endorsed by the publisher.

## Abbreviations

CEC: Cation exchange capacity
CAC: Cholesterol adsorption capacity
DW: Deionized water
DF: Dietary fiber
FT-IR: Fourier transformed infrared spectroscopy
IDF: Insoluble dietary fiber
W-IDF: Insoluble dietary fiber from wheat bran
LD: Loose density
NIAC: Nitrite ion adsorption capacity
ORC: Oil retention capacity
SEM: Scanning electron microscopy
SCAC: Sodium cholate adsorption capacity
SDF: Soluble dietary fiber
TBD: Tapped bulk density
TPC: Total phenolic content
WRC: Water retention capacity
WSC: Water swelling capacity
WB: Wheat bran
B-IDF: W-IDF modified by *B. subtilis*
A-IDF: W-IDF modified by *L. acidophilus*
P-IDF: W-IDF modified by *L. plantarum*
M-IDF: W-IDF modified by mixed lactic acid bacteria
C-IDF: W-IDF without microbial modification.
